# Body fat percentage is a better marker than body mass index for determining inflammation status in polycystic ovary syndrome 

**Published:** 2018-10

**Authors:** Andon Hestiantoro, Rachmat Dediat Kapnosa Hasani, Amalia Shadrina, Herbert Situmorang, Nurul Ilma, Raden Muharam, Kanadi Sumapraja, Budi Wiweko

**Affiliations:** *Department of Obstetrics and Gynecology, Faculty of Medicine Universitas Indonesia, Dr. Cipto Mangunkusumo Hospital, Jakarta, Indonesia*.

**Keywords:** Body fat, Body mass index, Inflammation, Polycystic ovary syndrome, Procalcitonin

## Abstract

**Background:**

Polycystic ovary syndrome (PCOS) is an endocrinopathic disorder most commonly experienced by women of reproductive age, and it is characterized by a low-grade chronic inflammatory condition. Excessive fat deposit has been long considered as an etiological factor in the pathogenesis of this inflammatory condition. Currently, body mass index (BMI) or percentage of body fat is used as a marker to assess the body fat composition of a person.

**Objective:**

To determine whether BMI or body fat percentage (BFP) can be used as a better marker for measuring inflammation related to body fat accumulation in polycystic ovary syndrome patients.

**Materials and Methods:**

This study took place at the Center for Reproductive Medicine, Yasmin Clinic, Cipto Mangunkusumo Hospital from January to December 2015. In this cross-sectional study, 32 reproductive age women with PCOS according to the Rotterdam criteria (2003) participated. Women with hyperandrogenism caused by non-classic congenital adrenal hyperplasia, pregnant and lactating women, etc., were excluded. Some variables such as BMI, clinical hyperandrogenism sign, BFP, and inflammatory markers were assessed and statistically analyzed.

**Results:**

From a total of 32 subjects of the study, BFP had a significant positive correlation with procalcitonin levels (r=0.35; p=0.048), while BMI did not (r=0.27; p=0.131).

**Conclusion:**

BFP can be used as a better marker for measuring inflammation related to body fat accumulation in PCOS subjects.

## Introduction

Polycystic ovarian syndrome (PCOS) is one of the most common endocrinopathic and metabolic disorders in woman during their reproductive years. The incidence of PCOS based on the Rotterdam criteria ranges between 14.6 and 19% depending on the population (1). PCOS causes symptoms such as infertility, menstrual disorder, and hyperandrogenism. Infertility and menstrual disorder are the most common symptoms and an estimated 75% of infertility is caused by ovulation dysfunction (2). Several factors play a role in the pathophysiology of PCOS, such as genetic, intrauterine, and environmental factors (3-5). Fat also plays an important role in the progression of the disease. A number of studies find that the majority of PCOS patients have an excess body fat level, even though several PCOS patients have a normal body fat level based on body mass index (BMI) (6, 7). 

Excessive fat tissue might manifest as fatty tissue hypertrophy, which causes a decrease in blood perfusion, which in turn leads to hypoxia. This also induces cell apoptosis, which causes a higher macrophage cell infiltration and enhances the secretion of pro-inflammatory cytokines. Therefore, excess fat tissue can be considered an etiological factor in the pathogenesis of low-grade chronic inflammation in PCOS (8). 

Low-grade chronic inflammation can be expressed if there is an enhancement in cytokines production such as C-reactive protein (CRP), tumor necrosis factor alpha (TNFα), procalcitonin (PCT), and interleukin-6 (IL-6), associated with the body’s immune response mechanism. PCT, the precursor of calcitonin, is a protein that is released into systemic circulation in response to systemic inflammation; it is also used as a severity level marker (9, 10). Serum PCT levels are very low or undetectable in the healthy state (10). Recently, elevated plasma PCT levels in the normal range have been associated with measures of obesity, insulin resistance, and other metabolic syndromes (10). In PCOS patients, excess body fat can cause an enhancement variety of inflammatory cytokines such as TNFα and IL-6 that can induce a PCT release from fat cells (11). Previously conducted studies also found that central body fat deposition and obesity in PCOS patients caused an elevation in inflammatory markers. Based on this relation, it can be concluded that PCT, as an inflammatory marker, might possess significant correlation with body fat. 

Therefore, this study aimed to determine the potential role of BFP and BMI as markers for measuring inflammation related to body fat composition in PCOS subjects.

## Materials and methods

This cross-sectional study was conducted in the Center for Reproductive Medicine, Yasmin Clinic, Cipto Mangunkusumo Hospital, Faculty of Medicine, Universitas Indonesia, involving patients with PCOS from January 2015 to December 2015. Our inclusion criteria were PCOS women aged 18-40 yr with at least two diagnoses under the Rotterdam criteria, that is, oligo-anovulation, clinical or biochemical hyperandrogenism, and a picture of the polycystic ovary in an ultrasonography examination (12). 

Those with hyperandrogenism caused by non-classic congenital adrenal hyperplasia, Cushing syndrome, uncontrolled thyroid disease, hyperprolactinemia, or other androgen-secreting tumors, those who consumed drugs that altered their endocrine, metabolism, or those who had an inflammation function three months prior to the examination, were pregnant or lactating, had acute or chronic infections, autoimmune disease or chronic inflammation, or those with a procalcitonin level of more than 500 pg/ml were excluded from the study. 

Initially, 43 women were enrolled in this study; 11 were excluded due to the exclusion criteria, so the final number of participants was 32 women. All patients underwent an examination to be diagnosed with PCOS. 

A history of infertility, menstrual cycle, menarche, family health, and previous medication was taken. All participants underwent physical exams that measured vital signs, general status, height and weight, signs of clinical hyperandrogenism, BMI, and BFP measurement using Bioelectrical Impedance Analysis (BIA) with the Tanita BC-601 Body Composition Monitor, which also calculates the total body water and fat mass. Ultrasonography examination was done to evaluate the ovary in terms of volume, size, and number of follicles. Then, for the participants who met the criteria, blood procalcitonin level was evaluated using an ABCAM human procalcitonin ELISA kit (AB221828, UK).

**Ethical considerations**

All participants had signed an informed consent form before inclusion in this study. The study protocol was approved by the ethics committee of the Faculty of Medicine, Universitas Indonesia, Jakarta, Indonesia (form No. 898/UN2.F1/ ETIK/2014).

**Statistical analysis**

MedCalc software (MedCalc, Version 13.3) and a Pearsons and Steigers test were used to compare two groups of the study. All p-values of less than 0.05 were considered significant.

## Results

A total of 32 subjects were recruited for this study. The characteristics of the study sample are described in Table I.

**Figure 1:**
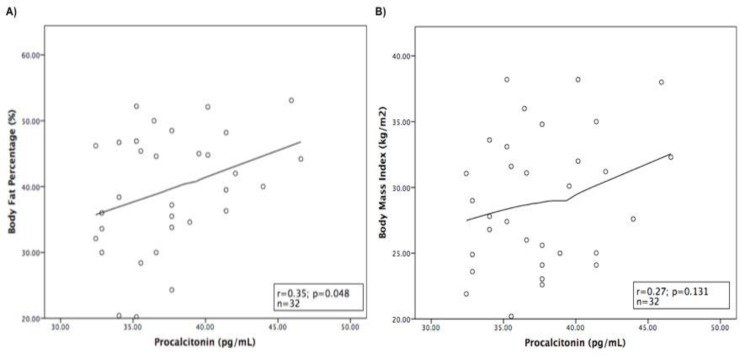
Scatter plot between BMI and BFP with procalcitonin level as an inflammation marker in PCOS subjects. A positive correlation between procalcitonin levels and BFP (A) was observed, while procalcitonin levels and BMI did not show any correlation (B)

Pearson analysis was used to determine the correlation between body fat composition (BMI and BFP) and procalcitonin levels. From the analysis, BMI showed no correlation (r=0.27; p=0.131) with procalcitonin level, while BFP had a moderate positive correlation (r=0.35) to procalcitonin levels, and it was statistically significant (p=0.048) (Figure 1).

To prove whether BFP might be a better marker for indicating the inflammation process in PCOS subjects compared to BMI, we conducted a comparison test between BMI coefficient correlation to procalcitonin and BFP to procalcitonin. In this study, BFP showed a moderate positive correlation with procalcitonin, while BMI did not show any correlation with procalcitonin (Table II). Based on those data, we can conclude that BFP has a better correlation than BMI toward procalcitonin level as an inflammatory marker in PCOS subjects.

**Table I:** Sample characteristics

**Characteristic**	**Description (n= 32)**
Age (yr)	29.19 ± 3.45
Height (cm)	158.93 ± 5.31
Weight (kg)	73.52 ± 13.51
Waist circumference (cm)	93.81 ± 8.86
BMI (kg/m^2^)	29.09 ± 5.11
BFP (%)	39.38 ± 9.04
FSH (mIU/ml)	6.83 ± 1.29
LH (mIU/ml)	9.58 ± 4.34
Prolactin (ng/ml)	10.63 ± 4.43
Testosterone (ng/ml)	39.61 ± 20.28
Procalcitonin (pg/ml)	37.56 ± 3.86

Values are presented as mean±SD.

BFP: Body fat percentage

BMI: Body mass index

FSH: Follicle stimulating hormone

LH: Luteinizing hormone.

**Table II:** Correlation of body fat composition with procalcitonin level

**Body fat composition parameter**	**r**	**p**
Body mass index	0.27	0.131
Body fat percentage	0.35	0.048

## Discussion

The aim of this study was to determine whether BMI or BFP as a marker for body fat composition show a correlation with procalcitonin as an inflammatory marker in subjects with PCOS.

PCT is a protein released into systemic circulation as a response to systemic inflammation (9). It strongly shows a positive correlation with other inflammatory biomarkers, such as high-sensitivity C-reactive protein (hs-CRP), white blood count (WBC), and neutrophil counts. Other studies revealed that PCT was an earlier, more specific and reliable marker than other inflammatory markers including TNF-, IL-6, or IL-8 because it rises faster in response to inflammation than CRP and decreases faster as inflammation subsides. PCT was also stated as a novel biomarker of chronic inflammatory activity of both body fat and PCOS (13).

Body fat composition plays a role in the pathophysiology of PCOS (11). Both BMI and BFP are parameters of body fat composition. BMI is most commonly used because it is easily measured. However, since body fat deposition turns out to have more clinical significance, especially in PCOS subjects, the assessment of the percentage of body fat seems to be more important than that of BMI. Body fat percentage or total fat mass were then used to reflect body fat composition, which can be measured by anthropometric examination or specific techniques including bioelectrical impedance analysis (BIA). Excess body fat can enhance the release of pro inflammatory cytokines, which can cause insulin resistance and hyperinsulinemia. Hyperinsulinemia can induce androgen production and decrease the sex hormone binding globulin level, which further causes visceral fat accumulation. That accumulation can lead to an endless cycle resulting in a worsening metabolism and hormonal disorder in PCOS patients (14).

Several previous studies report an increase of procalcitonin level as a low-grade chronic inflammation marker in PCOS patients compared to a normal woman (13, 15, 16). This finding was related to visceral obesity and central fat distribution, which showed excessive visceral fat played an important role in linking chronic inflammation with metabolic disorders, including PCOS (13). This study reported procalcitonin levels of about 37.56±3.86 pg/ml, which was higher than other PCOS studies, as follows: 14.3±0.42 pg/ml (16) 27.4±0.7 pg/ml (15) and 28.6±4.4 pg/ml (13). The differences of procalcitonin levels can be caused by varieties in blood sample types, measurement methods, and the reagent used for measuring procalcitonin levels.

The mean of BMI from the 32 subjects in this study was 29.095.11 kg/m^2^, which is classified as Grade 1 obese in the Asian classification. The mean BFP in this study was 39.389.04%, which was 35% higher than the American Society of Endocrinologists' recommendation for the ideal BFP in women. These results are in keeping with other PCOS studies that showed body fat composition in PCOS patients is higher than it is in the normal population (7, 17). BMI had no correlation with procalcitonin (r=0.27; p=0.131) in this study, which suggested that BMI was not an inflammation marker in PCOS subjects. A previous study stated that 42-88% of PCOS patients had BMI above the normal level (7). 

BFP had a moderate positive correlation with procalcitonin level, which was statistically significant (r=0.35; p=0.048). This finding is similar to that described by Puder and colleagues (r=0.47; p=0.001) (15). In this study, BFP had a better linearity than BMI in an enhancement of procalcitonin level as an inflammation marker in PCOS subjects (r=0.35; p=0.048 vs. r=0.27; p=0.131). This suggests that BFP is more accurate than BMI in describing body fat composition. This result is also similar with another study that reported that women with PCOS may have an excess BFP despite having a normal BMI (18, 19). Due to fat accumulation in several locations in PCOS patients, BMI had a weaker correlation in determining the inflammation process than BFP had. There is a probability of PCOS happening in women with a normal BMI.

This study also found that 31.25% of PCOS subjects had a normal BFP, which could occur with increasing body fat accumulation. Inflammation is related to the reproductive system and other cardiovascular disorders in PCOS patients. The small size of this study group could affect the findings, so the authors recommend further studies on inflammation markers in PCOS with more subjects. From this study, it can be concluded that BFP may have a greater effect in determining the inflammation process related to body fat composition in PCOS subjects (18, 19).

## Conclusion

Most of the PCOS subjects had excess body fat composition, with an average BMI of 29.09±5.11 kg/m^2^ (Obesity I) and BFP of 39.38±9.04% (normal value <35%). BMI had no correlation with procalcitonin levels, while BFP showed a moderate positive and a statistically significant correlation with procalcitonin. Therefore, we conclude that BFP might be a better marker in determining the inflammation marker in PCOS subjects.
